# Challenges in Treating Neonatal Acute Limb Ischemia: Conservative Management With Successful Limb Salvage

**DOI:** 10.1155/crvm/2424543

**Published:** 2025-03-24

**Authors:** Ayman Bsat, Leonard Lawandos, Sami Nabhani, Bassel Hafez, Fady Haddad

**Affiliations:** ^1^Department of General Surgery, American University of Beirut Medical Center, Beirut, Lebanon; ^2^Faculty of Medicine, American University of Beirut, Beirut, Lebanon; ^3^Division of Vascular and Endovascular Surgery, Department of Surgery, American University of Beirut Medical Center, Beirut, Lebanon

**Keywords:** arterial thrombosis, conservative management, limb salvage, neonatal acute limb ischemia, pediatric vascular emergencies

## Abstract

Acute limb ischemia (ALI) in neonates is a rare but critical condition often resulting from iatrogenic causes, such as arterial catheterization. This case highlights the unique challenges in diagnosing and managing ALI in this population, where evidence-based guidelines are scarce and treatment decisions rely heavily on expert consensus and extrapolation from adult data. We report the case of a premature neonate, born at 30 weeks' gestation, who developed ALI secondary to femoral artery thrombosis following arterial line insertion. The patient presented with bluish discoloration, diminished capillary refill, and absent distal pulses in the affected limb. Duplex ultrasonography confirmed occlusion of the right common femoral artery. Conservative management with anticoagulation and close observation was adopted by multidisciplinary consensus involving neonatology and vascular surgery. Despite signs of worsening ischemia and skin necrosis during therapeutic anticoagulation, the team opted to continue conservative management due to the high surgical risk posed by the patient's prematurity and multiple comorbidities. Over the next week, gradual clinical and imaging improvements were noted, including recanalization of the occluded artery and restoration of arterial flow, ultimately leading to successful limb salvage. This case underscores the importance of individualized, multidisciplinary decision-making in managing neonatal ALI. Conservative management with therapeutic anticoagulation can achieve favorable outcomes, even in cases with worsening ischemia, provided that close monitoring and optimal supportive care are ensured. It also highlights the need for further research to develop standardized pediatric guidelines for this rare but potentially devastating condition.

## 1. Introduction

Acute limb ischemia (ALI) is defined as a sudden reduction in arterial perfusion to an extremity, posing a significant threat to the viability of limb muscles and nerves if not reversed within 4–6 h [1, 2]. This condition necessitates prompt recognition and diagnostic evaluation and urgent therapeutic intervention to mitigate the risk of irreversible damage. Current published data address the adult population but lack information about the pediatric population, particularly infants. Most treatment decisions in caring for pediatric patients with ALI are based either on expert opinion or on small case reports in the literature. However, recent data recommend a distinct approach for pediatric patients compared to adults, as the underlying etiologies differ, necessitating heightened caution in the management of children, especially infants. The current approach to pediatric patients with ALI is initial conservative management with anticoagulation (AC) with interventions left for worsening clinical state [1].

## 2. Case Presentation

We present the case of a premature infant who developed ALI in the setting of femoral artery thrombosis as a complication of an arterial line insertion who was successfully treated conservatively. The parents of the patient provided verbal consent for the preparation and publication of this report. They were informed of its purpose and the measures taken to ensure anonymity and confidentiality. Identifying details have been omitted to safeguard the patient's privacy.

The patient was born by cesarean section as a product of in vitro fertilization (IVF) at 30 weeks and 4 days of gestation to a 45-year-old G2P0111 female for severe maternal preeclampsia and suspected HELLP syndrome, severe intrauterine growth restriction (IUGR), and absent end-diastolic flow in the umbilical artery on Doppler studies with nonreassuring fetal heart rate. Appropriate prenatal management was done, and the patient was admitted to the newborn intensive care unit (NICU) for respiratory distress syndrome of the newborn (RDS), suspected sepsis, and prematurity.

During the first month of life, the patient was treated in the NICU for thrombocytopenia, leukopenia, hyponatremia, ascites, and bronchopulmonary dysplasia. His stay was complicated with necrotizing enterocolitis (NEC) Stage II-A for which he was initially treated conservatively with antibiotics until his clinical state worsened with signs of intestinal obstruction. An urgent laparotomy with segmental resection of the jejunum with primary end–end anastomosis, repair of ascending colon, and creation of a diverting loop ileostomy was done at the age of 50 days. Prior to the surgery, an arterial line was placed using a 24-gauge catheter into the right femoral artery using the Seldinger technique with ultrasound guidance.

**Figure 1:**
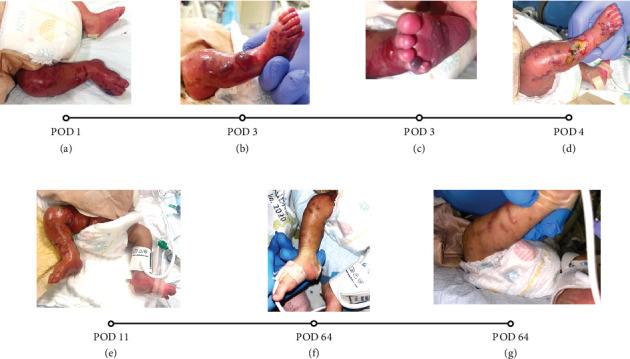
Clinical progression of the patient's right lower extremity, from the initial signs of ALI (a) to the development of threatened limb signs (b, c), and the healing stages of the ulcers (d–f). (a) Signs of critical limb ischemia detected postoperationally. (b) Bullae formation on POD 3, while on AC. (c) Bluish discoloration of the fourth right toe on POD 3, while on AC. (d) Aspirated bullae. (e) Increasing edema and erythema of the right LE compared to the left LE indicative of reperfusion syndrome. (f, g) Near-complete resolution of ALI signs, 2 months after initiation of AC.

**Figure 2:**
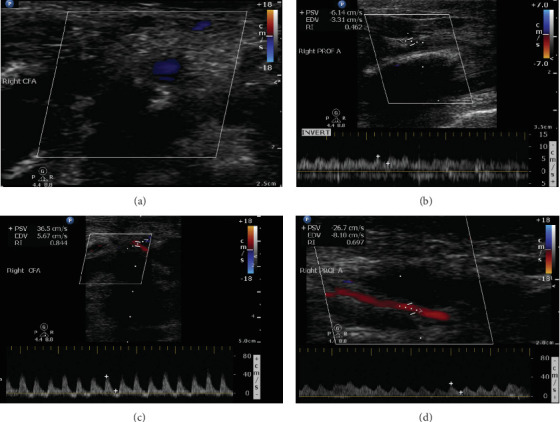
Lower extremity arterial duplex ultrasound (DUS) performed on the first day after ALI symptoms developed (a, b) and on POD 5, 5 days after initiation of anticoagulation (c, d). (a) Occluded right common femoral artery (CFA). (b) Patent right profunda artery (Prof A) with severely reduced sluggish flow pattern. (c) Recanalized right CFA filling from a pelvic collateral with biphasic waveform. (d) Patent right Prof A with a biphasic waveform.

Postoperatively, the patient was transferred back to the NICU. He was experiencing episodes of hypotension and was found to have metabolic acidosis and mild thrombocytopenia. On further examination, his right leg appeared slightly bluish with a decreased capillary refill compared to the left (Figure 1a). Doppler examination revealed a biphasic femoral signal on the right leg, but no Doppler signals were detected in the right dorsalis pedis (DP), posterior tibial (PT), or popliteal arteries. The femoral line was thus removed and warming of the lower extremity was initiated. Lower extremity arterial and venous duplex ultrasonography (DUS) was done and showed an occluded right common femoral artery with obstruction of flow distally (Figure 2a,b).

Therapeutic AC was initiated with heparin drip, targeting therapeutic activated partial thromboplastin time (aPTT) range between 60 and 85 s. On Postoperative Day (POD) 3, despite being on AC, physical examination revealed worsening signs of right lower extremity ischemia with signs of threatened limb in the setting of unstable blood pressure and low mean arterial blood pressure (MAP). Physical examination also showed multiple bullae with necrotic base over the right lower extremity and signs of skin necrosis over the tip of the right fourth and fifth toes (Figure 1b,c). Creatine phosphokinase (CPK) level was severely elevated, reaching a peak of 2525 IU/L on that day followed by a trend down to return to normal range 7 days later, with stable GFR and urine output. An urgent lower extremity DUS was thus done and revealed no change in the occluded right common femoral artery and absence of distal flow.

Given the worsening physical exam and persistence of the occlusion, a multidisciplinary meeting was held to decide on appropriate management. After carefully reviewing all available options with the parents, it was decided to continue conservative management with close clinical monitoring given the surgery's high risk of complications in the setting of the patient's status and comorbidities. Conservative management was focused on therapeutic AC, optimization of blood pressure, and optimization of patient's comorbidities.

AC was maintained as therapeutic heparin drip with the same aPTT target range until POD 10 when it was switched to enoxaparin, a low molecular weight heparin (LMWH), at a dose of 2 mg/kg of body weight every 12 h while targeting a therapeutic AC range of anti-factor Xa levels between 0.35 and 0.7 international units per milliliter (IU/mL) based on our lab normal ranges. We delayed transitioning to enoxaparin to allow appropriate healing of the affected leg before using the other leg for subcutaneous injections.

Over the following days, and in the setting of improved hemodynamics, clinical examination of the patient's lower extremity was improving as a faint femoral artery doppler signal was noted on POD 4, CPK level was trending down, and healing ulcers formed after aspiration of the previously noted bullae (Figure 1d). A repeat lower extremity DUS done on POD 5 revealed recanalization of the previously occluded right common femoral artery with improvement in the arterial flow in the right lower extremity (Figure 2c,d). On POD 11, severe foot swelling of the patient's right lower extremity was noted and was assumed to be in the phase of reperfusion syndrome after recanalization of the right arterial circulation with improving distal doppler signs (Figure 1e). Sequential lower extremity arterial DUS repeated at POD 9, 14, and 22 showed gradual improvement in the arterial circulation of the right lower extremity with arterial flow progressing from monophasic to biphasic to sharp biphasic waveform in all major arteries of the right lower extremity.

Given his associated comorbidities, including TPN-induced cholestasis and thrombocytopenia, anti-factor Xa was found to be subtherapeutic on multiple occasions despite increasing the dose gradually to 3.37 mg/kg every 12 h. However, in the setting of the clinical improvement seen both on arterial DUS and on physical examination and after consulting with the pediatric oncology team, AC was deemed to be appropriate despite the subtherapeutic anti-factor Xa levels and enoxaparin dose was finally switched to 3.37 mg/kg administered once daily on Day 25 of AC.

After 2 months of treatment with AC as detailed above, in parallel with handling all the other medical issues, a follow-up physical examination demonstrated significant improvements in motor power and sensory function (as reasonably possible at this age), as well as near-complete resolution of superficial ulcers (Figure 1f,g). However, it is important to note that the physical examination was limited due to the patient's age and inability to fully cooperate.

## 3. Discussion

ALI is an emergency condition that requires immediate diagnosis and treatment given its high risk of limb loss and mortality [1]. As per the European Society for Vascular Surgery (ESVS) 2020 Clinical Practice Guidelines on the Management of ALI, ALI is initially treated conservatively with further treatment determined by the clinical category of the limb proposed by Rutherford et al. [1, 3]. However, this recommendation has been extrapolated from adult ALI data, and the approach to managing pediatric patients remains unclear.

ALI is much less common in pediatric patients than in adult patients. In fact, Lim et al. observed the rate of ALI in pediatric patients to be 26 in every 100,000 pediatric hospital admission in one center [4]. This condition is even rarer in neonates as 2.4 out of every 1000 neonatal intensive care unit admissions were once reported to develop a thromboembolic event [2, 5]. Thus, literature on management of ALI in the pediatric population, particularly in the neonatal population, is scarce. Moreover, the etiological distribution of ALI varies in prevalence between different age groups [4]. These findings were mostly attributed to physiological differences, such as decreased oxygen demand due to lower muscle mass in infants' limbs, and healthier vasculature with a higher potential for developing collaterals compared to adults [4, 6, 7]. For instance, iatrogenic causes, most commonly catheter-related thrombosis, were found to be the most common etiologies in a cohort of pediatric patients; 84% of whom were aged less than 1 year [6, 7]. This pattern was observed in different retrospective cohorts and case series as well [1, 8].

Unlike ALI, femoral artery occlusion (FAO) is a more common and less severe complication of arterial catheterization among the pediatric population [6]. Our patient had multiple well-established risk factors for catheter-related arterial thrombosis, including prematurity, low birth weight, IUGR, sepsis, hypotension, thrombocytopenia, and prior major abdominal surgery [2]. Identifying these risk factors early is crucial, as they can help guide risk stratification and monitoring protocols in NICU settings.

As for treatment options, the available literature compared AC, thrombolysis, and surgical intervention for the treatment of ALI in infants. For instance, a cohort of 11 infants who developed ALI after arterial catheterization was treated by AC using either heparin or heparin followed by LMWH for a total period of 3–4 weeks [7]. Of these patients, most had a resolution of ALI with the exception of two patients who died during AC treatment period and one who required thrombectomy and fasciotomy at 48 h after AC treatment for ulceration and gangrene [7]. Also, a cohort of 25 infants diagnosed with ALI, mostly attributed to iatrogenic injury, found that AC was the treatment of choice in 80% of cases [8]. In this series, two patients required thrombolysis due to thrombus progression and one required an above the knee amputation at 6 weeks after diagnosis [8].

Similarly, a cohort of 151 patients who developed ALI, 86% of whom were less than 1 year of age, was reviewed to explore treatment options [6]. All patients with iatrogenic-induced ALI were successfully treated with AC using heparin or LMWH and eight patients were switched to thrombolytic therapy as no improvements were noted 24 h after initiation of AC [6]. Out of the eight patients treated with a thrombolytic, five responded completely, and the other three required a surgical intervention [6]. Likewise, the largest cohort study on ALI treatment in the pediatric population, involving 1576 patients with a mean age of 9.9 ± 7.1 years, compared surgical versus conservative management of ALI [4]. The study concluded that mortality was highest among infants, regardless of the treatment modality, and found no significant difference in survival outcomes between surgical and conservative approaches in this age group [4]. However, the surgical approach in infants was associated with a longer hospital stay and higher healthcare costs [4].

Literature reviews compiled by Sadat et al. and Cerbu et al. as well the ESVS 2020 Clinical Practice Guidelines on the Management of ALI reached similar conclusions on etiologies and management of ALI in neonates [1, 2, 9]. The suggested approach was to initiate AC immediately upon diagnosing ALI and transfer to a 24-h monitoring unit under the supervision of a multidisciplinary team [1, 2, 4, 6–9]. However, if significant motor or sensory deficits were present at presentation, thrombolysis or surgery would be considered [6]. As for follow-up recommendations, Kayssi et al. suggested a stepwise approach from thrombolysis followed by surgery in case of persistent obstruction, be it clinically or radiologically indicated [6]. In our case, despite signs of worsening ischemia—including skin necrosis, elevated CPK, and persistent arterial occlusion on ultrasound—our team opted to continue therapeutic AC rather than escalate to thrombolysis or surgical intervention. This decision was primarily influenced by the patient's high surgical risk due to extreme prematurity and comorbidities, aligning with prior studies that highlight the increased perioperative risks in neonates undergoing vascular interventions [4].

## 4. Conclusion

In conclusion, we have illustrated the case of a neonate who developed ALI as a result of arterial access and was successfully treated conservatively with AC and close clinical optimization despite worsening limb ischemia past Day 2 of AC. While conservative treatment was ultimately successful in our case, it remains unclear which neonates with ALI are most likely to benefit from noninterventional management versus those who require escalation of therapy. Literature reports suggest that early surgical or thrombolytic intervention should be considered if there is no clinical improvement within 24–48 h of AC initiation [6, 8]. However, in our patient, ischemic progression plateaued around POD 4, coinciding with a slow but steady improvement in Doppler signals and biochemical markers. This suggests that neonates may have a greater potential for collateral circulation development, allowing for earlier recanalization and potentially influencing future treatment algorithms.

## Data Availability

All data generated or analyzed during this study are included in this published article.
